# Lifestyle Factors and Metabolic Syndrome among Workers: The Role of Interactions between Smoking and Alcohol to Nutrition and Exercise

**DOI:** 10.3390/ijerph121215035

**Published:** 2015-12-16

**Authors:** Jui-Hua Huang, Ren-Hau Li, Shu-Ling Huang, Hon-Ke Sia, Yu-Ling Chen, Feng-Cheng Tang

**Affiliations:** 1 Occupational Health Center, Changhua Christian Hospital, Changhua 500, Taiwan; Juihua1025@gmail.com (J.-H.H.); qaz5410@yahoo.com.tw (Y.-L.C.); 2 Department of Psychology, Chung-Shan Medical University, Taichung 402, Taiwan; davidrhlee@yahoo.com.tw (R.-H.L.); shuling@csmu.edu.tw (S.-L.H.); 3 Room of Clinical Psychology, Chung-Shan Medical University Hospital, Taichung 402, Taiwan; 4 Division of Endocrinology and Metabolism, Changhua Christian Hospital, Changhua 500, Taiwan; 90279@cch.org.tw; 5 Department of Occupational Medicine, Changhua Christian Hospital, Changhua 500, Taiwan; 6 Department of Leisure Services Management, Chaoyang University of Technology, Taichung 413, Taiwan

**Keywords:** alcohol, health promotion, metabolic syndrome, nutrition and exercise behaviors, smoking, worker

## Abstract

This study aimed to investigate (1) relations of smoking and alcohol to metabolic syndrome (MetS) and its components, with nutrition and exercise controlled; and (2) interactions between smoking/alcohol and nutrition/exercise on MetS. This cross-sectional study enrolled 4025 workers. Self-reported lifestyles, anthropometric values, blood pressure (BP), and biochemical determinations were obtained. Among males, smoking significantly increased the risk of low high-density lipoprotein cholesterol (HDL-C), high triglyceride, abdominal obesity (AO), and MetS. Additionally, smoking showed significant interaction effects with nutrition on high BP, AO, and MetS; after further analysis, nutrition did not decrease above-mentioned risks for smokers. However, there was no significant interaction of smoking with exercise on any metabolic parameter. Alcohol increased the risk of AO, but decreased low HDL-C. It also showed an interaction effect with exercise on AO; after further analysis, exercise decreased AO risk for drinkers. Among females, alcohol significantly decreased the risk of high fasting blood glucose, but did not show significant interaction with nutrition/exercise on any metabolic parameter. In conclusion, in males, smoking retained significant associations with MetS and its components, even considering benefits of nutrition; exercise kept predominance on lipid parameters regardless of smoking status. Alcohol showed inconsistencies on metabolic parameters for both genders.

## 1. Introduction

The metabolic syndrome (MetS) is a clustering of abdominal obesity, elevated blood pressure (BP), low serum high-density lipoprotein cholesterol (HDL-C) levels, elevated serum triglycerides (TG), and impaired fasting blood glucose (FBG) [1]. MetS and its components, such as dyslipidemia and hypertension, increase the risk of cardiovascular disease [2]. The most important causes of MetS are aging, unhealthy diet, and sedentary lifestyle [3,4]. The characteristics of work, including job types, stress, and working hours are also associated with MetS [5,6,7]. Employees of several categories are therefore considered as a high-risk population in the development of MetS. In Taiwan, workplace health-promoting programs have been proposed to prevent the development of MetS. These strategies focus mainly on promoting workers’ physical activity and healthy eating [8]. 

Nutrition and exercise behaviors are important factors in the prevention of MetS and its components [3,9]. Lutsey *et al.* suggested that consumption of a Western dietary pattern, meat and fried foods promotes the incidence of MetS [3]. Reading nutrition labels showed an association with reducing the risk of MetS [10]. In addition, regular leisure-time physical activities prevent unhealthy weight gain [11]. Low level leisure physical activity raised the odds ratios of MetS [12]. Dietary and exercise behaviors were associated with obesity-specific quality of life and work productivity as well [13]. These studies stated that nutrition and exercise behaviors were confirmedly related to MetS and its components. 

Smoking and excessive alcohol drinking habits can also be contributing factors to developing MetS [14,15]. Cigarette smoking was considered to be related to MetS [16]. The smoking habit may increase the risk of lower HDL-C, higher TG, and abdominal obesity [17]. In addition, a higher drinking quantity and frequent binge drinking are associated with a higher prevalence of MetS, and are gender-specific [18]. Yoon *et al.* reported that alcohol consumption was inversely associated with the risk of low HDL-C, but showed an increasing dose-response relation between alcohol drinking and the risk of MetS [19]. In general, smoking and drinking habits are related to higher prevalence of MetS [20]. 

The individual influence of nutrition, exercise, smoking, and alcohol on the progression of MetS has been well studied, but little is known about the interactions of these lifestyle factors on the MetS and its components. A previous study indicated that smoking might have different detrimental effects on lipid-related cardiovascular disease in people with different grades of physical activity [21]. Breslow *et al.* also reported that alcohol-drinking patterns were associated with diet quality [22]. The associations between smoking/alcohol habits and nutrition/exercise behaviors may occur. However, only few investigators have analyzed the interactive effects of these lifestyle factors on the risk of MetS and its components in detail. Previous research stressed these interactive effects mainly on vascular diseases, such as stroke [23,24]; but studies regarding their influences on MetS were comparatively neglected. In Taiwan, a national survey in 2012 showed that about 32.7% of men and 4.3% of women were smokers. In addition, approximately 1.82% of the young adults and 4.82% of the middle-aged adults drank alcohol on a daily basis [25]. These issues should be considered. We thus investigated a series of lifestyle factors to clarify their interactions on MetS and its components among Taiwanese workers.

The interactive relationships among smoking, alcohol consumption, nutrition, exercise, and MetS were complicated. Further clarification of these associations is required. We proposed the hypotheses: (1) the associations between smoking or alcohol and MetS were positively significant, with nutrition and exercise controlled; (2) the interactions between smoking/alcohol and nutrition/exercise on MetS were significant as well. The aims of this study were: (1) to investigate the relations of smoking and alcohol habits to MetS itself and its components, when taking nutrition and exercise health behaviors into consideration; and (2) to explore the interactions between smoking/alcohol habits and nutrition/exercise behaviors on the development of MetS among male and female workers in Taiwan. 

## 2. Methods

### 2.1. Study Design

This study was conducted in 2012 using a cross-sectional research method with convenient sampling. Workers aged 20 years or over, from four companies, were recruited for the study. All of the participants took part in the study voluntarily. Personal information, nutrition, and exercise behaviors were obtained by a self-reported questionnaire. Blood biochemical data, such as FBG, TG, and HDL-C, and anthropometric measurements were collected by tests performed in medical laboratories and non-invasive physical examination. The present study was a component of the Taiwan Workplace Health Promotion Scheme, and was approved by an independent ethics committee, the Institutional Review Board of the Changhua Christian Hospital (Taiwan). 

### 2.2. Assessment of Nutrition and Exercise Health Behaviors

The data on nutrition and exercise behaviors were obtained using the subscales of Health-Promoting Lifestyle Profile II [26]. Nutrition behavior included nine items, and exercise behavior included eight items. These items were translated into traditional Chinese characters for the participants. In the present survey, the subscales of nutrition and exercise behaviors in Chinese have shown acceptable internal consistency, with Cronbach’s alpha of 0.78 and 0.85, respectively. Participants were asked to rate each item on a four-point Likert scale (1. Never, 2. Sometimes, 3. Often, and 4. Routinely). The mean score of each subscale, ranging from 1 to 4, was calculated from the total subscale scores divided by the number of response items. A higher score indicates a greater level of participation in health-promoting behaviors. 

### 2.3. Assessment of Smoking and Alcohol Drinking Habits

Information regarding smoking and alcohol drinking habits was collected from each question. Participants were asked to tick their statuses of smoking and alcohol drinking separately. Smoking habits were classified as following three conditions: non-smoking, occasional smoking, and daily smoking. For statistical purposes, workers who smoked occasionally or daily were classified as workers who smoked. In addition, workers were classified as non-alcohol drinking workers and alcohol drinking workers (including occasional and daily alcohol drinking). Of all the workers who smoked in this study, only seven of them were female. Therefore, the associations between smoking and dependent variables were not analyzed for the females.

### 2.4. Metabolic Parameters and Definition of MetS

MetS is defined as a co-occurrence of three out of five of the following medical conditions: elevated FBG, low HDL-C levels, high serum TG, elevated BP, and abdominal (central) obesity, according to the guidelines of the National Cholesterol Education Program [1]. According to the definition of the Health Promotion Administration, Ministry of Health and Welfare in Taiwan [27], FBG ≥ 100 mg/dL is considered as an impaired level. Ideal blood lipid levels are recommended as HDL-C ≥50 mg/dL among females and ≥ 40 mg/dL among males. TG < 150 mg/dL is optimal. Systolic BP <130 and diastolic BP < 85 are defined as normal levels of BP. Waist circumference (WC) ≥ 90 cm for men and ≥80 cm for women are considered as abdominal obesity. However, in the present study, the definition of MetS did not consider medical treatment or history of the disease.

### 2.5. Statistical Analysis

For statistical purposes, workers who smoked or drank alcohol were coded as 1 and non-smoking or non-drinking workers were coded as 0. Workers aged 40 or over were coded as 1 and workers aged under 40 were coded as 0. For categorical variables in the contingency table, data were presented in number (n) and percent (%). The chi-square test was used to examine the differences in smoking status, alcohol status and whether with MetS by gender. This test was also used to examine the relationships between smoking/alcohol status and MetS and each metabolic parameter (e.g., FBG < 100/≥100). For continuous variables, data were presented in mean ± SD, and comparisons of the means were analyzed by two-tailed *t*-test (two groups). Additionally, multiple logistic regression model with the “Enter” method was used to examine the individual relations of smoking and alcohol habits to MetS and its components separately for men and women; furthermore, interaction terms for lifestyle factors were added to this model to predict health outcomes. In this model, nutrition and exercise were first centered at their means respectively and then to form interaction terms with smoke or alcohol. This multiple logistic regression model comprised dependent variables including MetS and five metabolic parameters, and independent variables including age, lifestyle factors (smoking, alcohol drinking, nutrition, and exercise behaviors), and interaction terms for lifestyle factors. The analysis for women did not include smoking and interaction terms with it due to the very small number of female smokers. Data were presented with odds ratio (OR) and 95% confidence interval for OR (95% CIs). All statistical procedures were performed using SPSS 17.0 statistical software (SPSS Inc., Chicago, IL, USA); a *p*-value less than 0.05 was considered statistically significant. 

## 3. Results

### 3.1. Comparing the Characteristics of the Workers by Gender

In the present study, 5096 workers, aged 20 or over, were recruited as subjects from the four companies. Data with more than 25% of question items uncompleted were excluded to ensure the credibility of the results. In total, a final number of 4025 participants were enrolled. The average age of the participants was 43.1 ± 10.0 in the study. The comparisons of nutrition and exercise behaviors, metabolic parameters, MetS, smoking and alcohol of the participants by gender are summarized in Table 1. Male workers had significantly higher FBG, TG, systolic, and diastolic BP, WC, and percentage of MetS than the females did (*p* < 0.001). In addition, there were indications of gender differences in lifestyle factors, including nutrition behavior, smoking and alcohol habits (*p* < 0.001). Male workers had a lower nutrition behavior score, and higher percentages in cigarette smoking and alcohol drinking compared to the females.

**Table 1:** Comparing the characteristics of the workers by gender.

Variables	Total (*n* = 4025)	Male (*n* = 3294)	Female (*n* = 731)	
Continuous variables (Mean ± SD)				*p* for *t*-test
Age (y)	43.1 ± 10.0	42.6 ± 10.3	45.6 ± 7.6	<0.001
Exercise behavior score	1.96 ± 0.56	1.96 ± 0.56	1.96 ± 0.54	0.884
Nutrition behavior score	2.47 ± 0.44	2.43 ± 0.43	2.66 ± 0.43	<0.001
Metabolic parameters				
FBG (mg/dL)	93.4 ± 18.8	94.2 ± 19.6	89.7 ± 14.6	<0.001
HDL-C (mg/dL)	52.8 ± 12.6	50.8 ± 11.7	61.3 ± 13.0	<0.001
TG (mg/dL)	133.7 ± 101.1	139.1 ± 105.4	109.1 ± 74.2	<0.001
Systolic BP (mmHg)	123.1 ± 15.5	124.5 ± 14.9	116.6 ± 16.7	<0.001
Diastolic BP (mmHg)	78.7 ± 11.3	79.1 ± 11.3	76.9 ± 10.9	<0.001
WC (cm)	82.0 ± 9.5	83.9 ± 8.7	73.6 ± 8.4	<0.001
Categorical variables (n (%))				*p* for chi-square test
MetS				
<3 components	3347 (83.2)	2698 (81.9)	649 (88.8)	<0.001
≥3 components	677 (16.8)	595 (18.1)	82 (11.2)	
Smoking				
with	905 (22.3)	898 (27.0)	7 (1.0)	<0.001
without	3150 (77.7)	2425 (73.0)	725 (99.0)	
Alcohol				
with	1833 (45.2)	1694 (51.0)	139 (19.0)	<0.001
without	2218 (54.8)	1627 (49.0)	591 (81.0)	

Notes: FBG: fasting blood glucose; HDL-C: high-density lipoprotein cholesterol; TG: triglyceride; BP: blood pressure; WC: waist circumference; MetS: metabolic syndrome.

### 3.2. Comparing Metabolic Parameters by Smoking and Alcohol Habits

Differences in metabolic parameters by smoking and alcohol habits are shown in Table 2. The workers with smoking had a higher proportion of low HDL-C (21.9% *vs.* 14.1%, *p* < 0.001), high TG (36.4% *vs.* 25.5%, *p* < 0.001) and MetS (22.0% *vs.* 15.3%, *p* < 0.001), when compared with the non-smoking workers. In addition, the workers who drank alcohol had a higher proportion of high FBG (19.7% *vs.* 17.0%, *p* = 0.023), high TG (29.8% *vs.* 26.4%, *p* = 0.016), and increased WC (24.4% *vs.* 21.2%, *p* = 0.016), when compared with the workers who did not drink alcohol. The difference of MetS by alcohol drinking had a marginal tendency toward statistical significance (*p* = 0.053).

**Table 2:** Comparing metabolic parameters by smoking and alcohol habits.

Variables	Smoking			Alcohol Drinking		
	With, n (%)	Without, n (%)	*p*	With, n (%)	Without, n (%)	*p*
FBG (mg/dL)						
<100	713 (79.7)	2579 (82.4)	0.057	1456 (80.3)	1831 (83.0)	0.023
≥100	182 (20.3)	549 (17.6)		358 (19.7)	374 (17.0)	
n	895	3128		1814	2205	
HDL-C (mg/dL)						
<40 for men or <50 for women	198 (21.9)	444 (14.1)	<0.001	270 (14.8)	370 (16.7)	0.091
≥40 for men or ≥50 for women	707 (78.1)	2700 (85.9)		1560 (85.2)	1845 (83.3)	
n	905	3144		1830	2215	
TG (mg/dL)						
<150	576 (63.6)	2346 (74.5)	<0.001	1287 (70.2)	1633 (73.6)	0.016
≥150	329 (36.4)	804 (25.5)		546 (29.8)	585 (26.4)	
n	905	3150		1833	2218	
BP (mmHg)						
Systolic BP < 130/Diastolic BP < 85	536 (59.2)	1854 (58.9)	0.842	1064 (58.0)	1327 (59.8)	0.251
Systolic BP ≥ 130/Diastolic BP ≥ 85	369 (40.8)	1296 (41.1)		769 (42.0)	891 (40.2)	
n	905	3150		1833	2218	
WC (cm)						
<90 for men or <80 for women	685 (75.7)	2453 (77.9)	0.167	1386 (75.6)	1748 (78.8)	0.016
≥90 for men or ≥80 for women	220 (24.3)	697 (22.1)		447 (24.4)	470 (21.2)	
n	905	3150		1833	2218	
MetS						
<3 components	698 (78.0)	2643 (84.7)	<0.001	1484 (81.9)	1855 (84.2)	0.053
≥3 components	197 (22.0)	479 (15.3)		327 (18.1)	347 (15.8)	
n	895	3122		1811	2202	

Notes: FBG: fasting blood glucose; HDL: high-density lipoprotein; TG: triglycerides; BP: blood pressure; WC: waist circumference; MetS: metabolic syndrome.

### 3.3. Interactions between Smoking/Alcohol Habits and Exercise/Nutrition Behaviors on the Risk of Metabolic Parameters

The results of smoking and alcohol habits and their separate interactions with nutrition and exercise behaviors in relation to the risks of metabolic parameters are shown in Table 3.

For male participants, a smoking habit showed higher risks in all of metabolic parameters. The risks even reached a significant level in low HDL-C (OR = 2.01, 95% CI 1.61–2.52, *p* < 0.001), high TG (OR = 1.62, 95% CI 1.35–1.94, *p* < 0.001), and increased WC (OR = 1.22, 95% CI 1.01–1.49, *p* = 0.042). In addition, the interaction effect of a smoking habit and nutrition behavior on high BP showed a statistical significance (OR = 1.06, 95% CI 1.00–1.11, *p* = 0.033). Meaning, nutrition behavior on the risk of developing high BP for smoking male workers relative to that of non-smoking male workers was 1.06. For male non-smoking workers, nutrition behavior significantly decreased the risk of elevated BP (OR = 0.97, 95% CI 0.94–1.00, *p* = 0.036). After further analysis, nutrition behavior did not significantly decrease the risk of high BP for male workers with smoking (OR = 1.06 × 0.97 = 1.03, 95% CI 0.94–1.11, *p* = 0.512). The interaction effect of smoking and nutrition on increased WC (OR = 1.08, 95% CI 1.02–1.14, *p* = 0.011) also showed significant results. For non-smoking workers, the OR of nutrition behavior for increased WC was 0.99. The OR was not significantly different from unity. After further analysis for workers with smoking, nutrition behavior was observed to constitute a non-significant risk of experiencing increased WC (OR = 1.08 × 0.99 = 1.07, 95% CI 0.98–1.17, *p* = 0.079). Smoking habit did not show any significant interaction with exercise behavior on metabolic parameters, though exercise was observed to have individual significant effect on reducing the risk of low HDL-C (OR = 0.95, 95% CI 0.92–0.99, *p* = 0.010) and high TG (OR = 0.96, 95% CI 0.93–0.98, *p* = 0.002). It implies exercise behavior kept the predominant benefits on lipid parameters regardless of smoking status. 

**Table 3:** Interactions between smoking/alcohol habits and exercise/nutrition behaviors on the risk of metabolic parameters.

Variables	FBG (mg/dL) ≥100		HDL-C (mg/dL) <40 for Men or <50 for Women		TG (mg/dL) ≥150		BP (mm Hg) SBP ≥130 and DBP ≥85		WC (cm) ≥90 for Men or ≥80 for Women	
	OR (95% CI)	*p*	OR (95% CI)	*p*	OR (95% CI)	*p*	OR (95% CI)	*p*	OR (95% CI)	*p*
Man										
Constant	0.20 (0.17–0.24)	<0.001	0.22 (0.19–0.27)	<0.001	0.28 (0.24–0.33)	<0.001	0.41 (0.35–0.47)	<0.001	0.21 (0.18–0.25)	<0.001
Age (≥40/<40)	1.24 (0.03–1.49)	0.021	0.55 (0.44–0.67)	<0.001	1.76 (1.50–2.07)	<0.001	2.99 (2.56–3.48)	<0.001	1.42 (1.20–1.70)	<0.001
Nutrition	1.00 (0.97–1.04)	0.891	1.03 (0.98–1.07)	0.215	1.01 (0.98–1.04)	0.568	0.97 (0.94–1.00)	0.036	0.99 (0.96–1.03)	0.765
Exercise	0.99 (0.95–1.02)	0.354	0.95 (0.92–0.99)	0.010	0.96 (0.93–0.98)	0.002	1.02 (1.00–1.05)	0.115	0.97 (0.94–1.00)	0.090
Smoke (with/without)	1.06 (0.86–1.31)	0.589	2.01 (1.61–2.52)	<0.001	1.62 (1.35–1.94)	<0.001	1.09 (0.92–1.31)	0.318	1.22 (1.01–1.49)	0.042
Alcohol (with/without)	1.19 (1.00–1.42)	0.056	0.72 (0.58–0.89)	0.002	0.98 (0.84–1.14)	0.777	1.06 (0.92–1.23)	0.431	1.22 (1.03–1.44)	0.023
Smoke × Nutrition	1.00 (0.94–1.06)	0.872	1.05 (0.98–1.12)	0.157	1.02 (0.97–1.08)	0.377	1.06 (1.00–1.11)	0.033	1.08 (1.02–1.14)	0.011
Smoke × Exercise	1.00 (0.96–1.05)	0.651	1.01 (0.95–1.06)	0.833	0.98 (0.94–1.02)	0.369	0.99 (0.94–1.03)	0.474	1.05 (1.00–1.10)	0.060
Alcohol × Nutrition	1.02 (0.96–1.07)	0.560	0.97 (0.91–1.03)	0.301	0.98 (0.94–1.03)	0.472	1.02 (0.98–1.07)	0.284	1.03 (0.98–1.08)	0.283
Alcohol × Exercise	1.00 (0.96–1.05)	0.847	1.01 (0.96–1.07)	0.708	1.02 (0.98–1.06)	0.456	0.98 (0.95–1.02)	0.324	0.95 (0.91–0.99)	0.018
Woman										
Constant	0.06 (0.03–0.13)	<0.001	0.22 (0.14–0.34)	<0.001	0.12 (0.07–0.22)	<0.001	0.13 (0.07–0.22)	<0.001	0.21 (0.13–0.33)	<0.001
Age (≥40/<40)	3.06 (1.28–7.29)	0.012	1.14 (0.71–1.83)	0.601	1.84 (1.02–3.32)	0.044	4.07 (2.30–7.20)	<0.001	1.53 (0.94–2.51)	0.091
Nutrition	0.94 (0.88–1.01)	0.076	1.02 (0.96–1.07)	0.578	0.94 (0.88–1.00)	0.036	0.98 (0.93–1.03)	0.341	0.94 (0.89–0.99)	0.023
Exercise	1.05 (0.99–1.12)	0.125	0.99 (0.94–1.04)	0.727	0.99 (0.94–1.05)	0.828	0.99 (0.94–1.03)	0.581	0.99 (0.94-1.040	0.561
Alcohol (with/without)	0.21 (0.07–0.65)	0.006	1.17 (0.71–1.92	0.540	0.74 (0.40–1.37)	0.337	0.75 (0.46–1.21)	0.239	0.92 (0.56–1.51)	0.739
Alcohol × Nutrition	1.17 (0.92–1.47)	0.196	0.97 (0.86–1.10)	0.675	1.02 (0.87–1.19)	0.815	1.00 (0.88–1.13)	0.983	1.00 (0.87–1.15)	0.993
Alcohol × Exercise	0.89 (0.72–1.10)	0.277	0.96 (0.85–1.07)	0.423	0.87 (0.75–1.02)	0.084	1.02 (0.92–1.14)	0.668	0.94 (0.84–1.07)	0.355

Notes: FBG: fasting blood glucose (Including 3284 male and 729 female subjects in this multiple logistic regression); HDL-C: high-density lipoprotein cholesterol (3309 male and 730 female study subjects); TG: triglyceride (3315 male and 730 female study subjects); BP: blood pressure (3315 male and 730 female study subjects); WC: waist circumference (3315 male and 730 female study subjects).

In male workers, alcohol drinking significantly increased the risk of increased WC (OR = 1.22, 95% CI 1.03–1.44, *p* = 0.023), but it decreased the risk of low HDL-C (OR = 0.72, 95% CI 0.58–0.89, *p* = 0.002). In addition, the interaction effect of alcohol drinking habit and exercise behavior on increased WC displayed a statistical significance (OR = 0.95, 95% CI 0.91–0.99, *p* = 0.018). For workers who did not drink alcohol, exercise behavior did not significantly decrease the risk of increased WC; however, exercise behavior significantly decreased the risk of increased WC (OR = 0.95 × 0.97 = 0.92, 95% CI 0.86–0.99, *p* = 0.004) for workers with alcohol drinking. Compared with workers who did not drink alcohol, exercise behavior significantly decreased the risk of increased WC in workers with alcohol drinking. Besides, alcohol habits did not show any significant interaction with nutrition behavior on any metabolic parameter.

For female participants, alcohol drinking on reducing high FBG reached a significant level (OR = 0.21, 95% CI 0.07–0.65, *p* = 0.006). However, alcohol drinking did not show significant interaction effects with nutrition or exercise behavior on the risk of metabolic parameters.

### 3.4. Interaction between Smoking/Alcohol Habits and Exercise/Nutrition Behaviors on the Risk of MetS

The results of smoking and alcohol habits and their separate interactions with nutrition and exercise behaviors in relation to the risks of MetS are shown in Table 4.

For male participants, a smoking habit showed a significantly higher risk in MetS (OR = 1.64, 95% CI 1.33–2.02, *p* < 0.001). The interaction effect of smoking and nutrition on MetS (OR = 1.07, 95% CI 1.01–1.14, *p* = 0.028) also showed a significant result. For non-smoking workers, the OR of nutrition behavior for MetS was 0.96. After further analysis for workers who smoked, nutrition behavior showed a non-significant risk of MetS (OR = 1.07 × 0.96 = 1.03, *p* = 0.357). In addition, a smoking habit did not show any significant interaction with exercise behavior on MetS. On the other hand, an alcohol habit did not show any significant interaction with nutrition behavior on MetS for male participants.

For female participants, alcohol drinking did not show significant interaction effects with nutrition or exercise behavior on the risk of MetS. The analysis for women did not include smoking due to the very small number of female participants with smoking.

**Table 4:** Interactions between smoking/alcohol habits and exercise/nutrition behaviors on the risk of MetS.

Variables	MetS	*p*
	OR (95% CI)	
Man		
Constant	0.13 (0.11–0.16)	<0.001
Age (≥40/<40)	1.79 (1.47–2.17)	<0.001
Nutrition	0.96 (0.93–1.00)	0.068
Exercise	0.98 (0.94–1.01)	0.170
Smoke (with/without)	1.64 (1.33–2.02)	<0.001
Alcohol (with/without)	1.06 (0.88–1.28)	0.530
Smoke × Nutrition	1.07 (1.01–1.14)	0.028
Smoke × Exercise	1.00 (0.95–1.05)	0.977
Alcohol × Nutrition	1.05 (0.99–1.11)	0.125
Alcohol × Exercise	0.98 (0.94–1.03)	0.498
Woman		
Constant	0.07 (0.04–0.14)	<0.001
Age (≥40/<40)	2.37 (1.14–4.96)	0.021
Nutrition	0.90 (0.84–0.97)	0.005
Exercise	0.98 (0.92–1.05)	0.598
Alcohol (with/without)	0.67 (0.33–1.39)	0.284
Alcohol × Nutrition	0.98 (0.79–1.20)	0.822
Alcohol × Exercise	0.89 (0.73–1.08)	0.233

Note: MetS: metabolic syndrome (Including 3315 male and 730 female subjects in this multiple logistic regression).

## 4. Discussion

The present study provides evidence of lifestyle interactions with regard to MetS and its components among Taiwanese workers. Statistical analysis of the smoking habit was carried out for only male participants because the number of female participants who smoked was small. The results in this study found that smoking increased the risk of MetS and its components among male workers. Particularly, there were significant associations with HDL-C, TG, WC and MetS. Moreover, smoking showed interaction effects with nutrition behavior on BP, WC, and MetS. However, alcohol drinking showed inconsistent associations with MetS and its components, regardless whether they were males or females. For male workers, an alcohol habit increased the risk of increased WC, but decreased the risk of low HDL-C. In addition, alcohol showed an interaction effect with exercise behavior on WC. For female workers, an alcohol habit significantly decreased the risk of high FBG. However, alcohol drinking did not have a significant interaction effect with exercise or nutrition behavior on the risk of MetS.

Smoking may raise sympathetic activity and increase the circulating cortisol, catecholamines, vasopressin, and growth hormone levels [28,29]. Therefore, smoking has been considered as playing a causal role in the development of MetS [29]. Slagter *et al.* reported that smoking was significantly associated with lower HDL-C, higher TG, and abdominal obesity [17]. Calo *et al.* also reported that there was an increased risk of MetS among current smokers, especially those with excess consumption [30]. Indeed, our findings, which indicated that smoking increased the risk of low HDL-C, high TG, abdominal obesity, and MetS in male workers, were consistent with the results in other studies.

In the present study, nutrition health behavior significantly decreased the risk of elevated BP in male workers non-smoking. However, the interaction effect of smoking and nutrition behavior was significant with a higher odd ratio on elevated BP. That is to say, nutrition behavior among smoking and non-smoking males had different associations with developing elevated BP. In addition, the result of further analysis for male workers who smoked showed that nutrition behavior did not significantly decrease the risk of high BP. In previous research, consumption of an unhealthy diet was associated with elevated BP [31]. Conversely, good nutrition behavior showed an association in reducing the risk of high BP [32]. Furthermore, smoking was also found to be an important risk factor for developing high BP [33]. Our results implied that the benefit of nutrition behavior might not surpass the adverse effects of smoking in regard to the impact on high BP in male workers. A smoking habit should therefore be taken into consideration when designing workplace health promotion programs to prevent the development of elevated BP. The mechanism of the interaction of smoking and nutrition behavior on BP also deserves further study.

The influences of alcohol drinking on the components of MetS exhibit both positive and negative results [34]. The association between alcohol drinking and development of MetS is stronger among red wine drinkers due to polyphenoles contained in red wine. Polyphenoles may raise the activity of endothelial nitric oxide synthase (eNOS), which plays a key role in the development of MetS. Promoting eNOS activity, which involves the transport of HDL-C, may elucidate the mechanism of the elevated plasma levels of HDL-C in response to alcohol. Conversely, downregulating eNOS activity may result in the incidence of insulin resistance, high BP, and abnormal blood lipid levels [34]. Yoon *et al.* reported that alcohol consumption had a negative association with the risk of low HDL-C, but also that there was an increasing dose-response relation between alcohol consumption and the risk of MetS [19]. In addition, research exploring gender-specific relationships between alcohol patterns and the prevalence of MetS has been carried out. Lee indicated that excessive drinking frequency was dose-dependently associated with elevated TG, hyperglycemia, elevated BP, and abdominal obesity in men, and with hyperglycemia and elevated BP in women [18]. Another study showed that higher alcohol consumption raised the risk of abdominal obesity in men only [35]. Our data also showed that the associations between alcohol drinking and components of MetS were controversial and gender differential. In this study, an alcohol habit decreased the risk of low HDL-C, and increased the risk of abdominal obesity in male workers; alcohol habit decreased the risk of high FBG in female workers. 

The interaction between alcohol and either nutrition or exercise behavior was seldom discussed in previous studies. The amount and type of alcohol consumed was considered as playing important roles in enhancing or inhibiting the development of MetS [36,37]. A recent study showed medium-high risk alcohol consumption mainly affected males, and was associated with increased risk of hypertension; among females, low risk consumption was associated with a lower prevalence of certain components of the MetS, including obesity and hypertension [38]. Wang *et al.* also reported that light to moderate alcohol consumption was found to be protective regarding the risks of obesity or being overweight among females [39]. In our study, the interaction effects of alcohol drinking and either nutrition or exercise behavior on MetS and its components were not significant, except for WC among male workers. More detailed information regarding the amount and type of alcohol consumed is recommended for relevant research in the future. Notably, the benefit of exercise behavior can be found in the impact on WC in alcohol drinking male workers. For this group of workers, to prevent the development of abdominal obesity, exercise behavior should therefore be taken into consideration when designing workplace health promotion program.

Females usually have a lower rate of alcohol drinking or smoking than males in Taiwan and several countries [25,38,40]. A national survey in Taiwan showed that approximately 29.6% of male employees and 3.1% of female employees were smokers; the occurrences of alcohol drinking were 37.8% for male employees and 13.3% for female employees. Also, the prevalence of MetS among male workers was significantly higher than among female workers in this study. In particular, the relations of smoking and alcohol drinking to the development of MetS were gender-specific. Therefore, gender difference should be an issue when establishing policy to prevent MetS and in designing workplace health-promoting programs.

Although this study has provided some insights into the interaction effects of nutrition/exercise and smoking/alcohol on the risk of MetS and its components, it has several limitations. First, there was about 21% of missing data in the present study. We were not sure whether they were missing at random (MAR). The results should therefore be interpreted carefully. Second, the assessment of lifestyle was highly dependent on the self-reported questionnaire. Overestimation, underestimation, or poor recall may produce confounded results. Third, our analyses did not consider frequency, consumption, and specific types of alcohol. Fourthly, the definition of metabolic syndrome did not consider the history of the disease or the effectiveness of medical treatment, and thus the classification of metabolic syndrome may have a bias. This study focused on the participants’ current state of MetS. Finally, it was a cross-sectional study and could not establish the causal direction of the impacts of smoking and alcohol habits on the risk of developing MetS and its components. The inference of the findings should therefore be applied with caution. Further studies adopting a longitudinal study design, with objective tools to more accurately assess frequency, quantity, and types of alcohol consumption, as well as lifestyle factors are recommended.

## 5. Conclusions

Male workers with smoking habits showed higher risks on MetS and its components. Moreover, smoking habits showed a significant interaction effect with nutrition behavior on the risk of high BP, abdominal obesity, and MetS in male workers, though its interaction with exercise was not significant. In general, taking the benefits of nutrition behavior into further consideration, smoking habits still retained significant relations with MetS and its components. Exercise kept the predominant benefits on lipid parameters regardless of smoking status. Alcohol drinking showed inconsistent associations with the development of MetS for both males and females, although, for male workers with alcohol habits, maintaining an exercise routine could be tried to decrease the risk of abdominal obesity. It is therefore recommended that workplace health promotion practitioners should develop more specific strategies to target male workers with smoking habit or poor exercise behavior.
